# Can you hear my age? Influences of speech rate and speech spontaneity on estimation of speaker age

**DOI:** 10.3389/fpsyg.2015.00978

**Published:** 2015-07-17

**Authors:** Sara Skoog Waller, Mårten Eriksson, Patrik Sörqvist

**Affiliations:** ^1^Department of Social Work and Psychology, University of GävleGävle, Sweden; ^2^Department of Building, Energy and Environmental Engineering, University of GävleGävle, Sweden

**Keywords:** age estimation, speech perception, speech rate, cognitive speech processing, speech spontaneity

## Abstract

Cognitive hearing science is mainly about the study of how cognitive factors contribute to speech comprehension, but cognitive factors also partake in speech processing to infer non-linguistic information from speech signals, such as the intentions of the talker and the speaker’s age. Here, we report two experiments on age estimation by “naïve” listeners. The aim was to study how speech rate influences estimation of speaker age by comparing the speakers’ natural speech rate with increased or decreased speech rate. In Experiment 1, listeners were presented with audio samples of read speech from three different speaker age groups (young, middle aged, and old adults). They estimated the speakers as younger when speech rate was faster than normal and as older when speech rate was slower than normal. This speech rate effect was slightly greater in magnitude for older (60–65 years) speakers in comparison with younger (20–25 years) speakers, suggesting that speech rate may gain greater importance as a perceptual age cue with increased speaker age. This pattern was more pronounced in Experiment 2, in which listeners estimated age from spontaneous speech. Faster speech rate was associated with lower age estimates, but only for older and middle aged (40–45 years) speakers. Taken together, speakers of all age groups were estimated as older when speech rate decreased, except for the youngest speakers in Experiment 2. The absence of a linear speech rate effect in estimates of younger speakers, for spontaneous speech, implies that listeners use different age estimation strategies or cues (possibly vocabulary) depending on the age of the speaker and the spontaneity of the speech. Potential implications for forensic investigations and other applied domains are discussed.

## Introduction

Cognitive hearing science is mainly about how cognitive factors contribute to speech comprehension (Arlinger et al., 2009), such as how working memory (Rönnberg et al., 2013) and long-term memory (Sörqvist et al., 2014) supports speech comprehension in adverse listening conditions, and how the mind tries to predict upcoming information in the unfolding speech stream (Bendixen et al., 2009). However, cognitive factors can also partake to extract non-linguistic information from speech signals. Indexical information of a person (see Harnsberger et al., 2008) such as gender, age, height, and weight can be extracted with some certainty from voice alone (Krauss et al., 2002; Hughes and Gallup, 2008). This paper investigates this relatively understudied form of cognitive speech processing. Specifically, it explores in two experiments how variations in one aspect of the speech signal—speech rate—influence age estimation. The first experiment is based on read speech whereas the second is based on spontaneous speech. Most previous research on age estimates from voice has been done on read speech (Ptacek and Sander, 1966; Ramig and Ringel, 1983; Huntley et al., 1987; Shipp et al., 1992; Braun, 1996; Braun and Cerrato, 1999; Cerrato et al., 2000; Harnsberger et al., 2008; Torre and Barlow, 2009). However, most communication come about spontaneously why age estimates from spontaneous communication is of obvious interest. The results may have implications for various applied areas such as acting (e.g., Werner, 1996), speech synthesis (e.g., Schötz, 2006), speech and hearing disorders (e.g., Harnsberger et al., 2008) and forensic investigations (e.g., Yarmey et al., 1996).

When inferring the age of the speaker from voice, a listener may rely on various cues to infer the age of the speaker from the physical attributes of the voice as well as the contents (linguistic attributes) of what is being said (Moyse, 2014). For example, older adults produce less fluent and less complex speech in comparison with younger adults (Kemper et al., 2003). Examples of physical speech attributes that change with age is fundamental frequency, amount of shimmer and speech rate. The fundamental frequency of the voice changes at puberty and during the transition into adulthood (Hughes and Rhodes, 2010) and correlates with other physiological changes as people gets older and the amount of shimmer is found to increase (Ramig and Ringel, 1983; Xue and Hao, 2003). Whilst most age-related changes in the fundamental frequency take place prior to adulthood (Huber et al., 1999; Lee et al., 1999; Amir and Biron-Schental, 2004), speech rate continues to change considerably after adulthood. As people get older, speech rate decreases (Linville, 2001; Brückl and Sendlmeier, 2003; Schötz, 2006). All age related changes of speech may not be used in an age estimation task, but speech rate seems of greatest relevance (Harnsberger et al., 2008). People may hence incidentally learn the association between speech rate and age of speakers in their everyday interactions with others. If these associations have been learned and if speech rate is used as a cue to age estimates, manipulations of speech rate should influence age estimates of adult speakers.

The accuracy of age estimates based on voice is poor when compared to age estimates from faces (Rhodes, 2009; Moyse, 2014). Although the magnitude of correlations between age estimates and the chronological age of the speaker is typically high (Shipp and Hollien, 1969; Huntley et al., 1987; Neiman and Applegate, 1990; Braun, 1996; Cerrato et al., 2000; Brückl and Sendlmeier, 2003), the age of young speakers is systematically overestimated and the age of older speakers is systematically underestimated (Shipp and Hollien, 1969; Hollien and Tolhurst, 1978; Huntley et al., 1987; Braun, 1996; Braun and Cerrato, 1999; Cerrato et al., 2000; Brückl and Sendlmeier, 2003). The cause of this effect may simply be that, when cues to the accurate estimate are scarce, the best strategy would be to guess on an age estimate close to the middle of the possible age range to minimize error (Fahsing et al., 2004). The resulting biases are typical of research on estimation of person characteristics. In the present study, the accuracy of the age estimates is also used as a control of task difficulty. Extant research shows that age estimation of younger individuals is easier (i.e., has greater accuracy) than age estimation of older individuals (Rhodes, 2009; Vestlund et al., 2009; Moyse, 2014). We explored task difficulty in the context of accuracy estimates, because difference in task difficulty may be informative when the effects of speech rate on over- and underestimates are interpreted. Here, accuracy is defined as the absolute difference between the age estimate and the chronological age of the speaker, whereas over- and underestimates are calculated by taking the signed difference between the age estimate and the chronological age of the speaker (Vestlund et al., 2009). When averaged across estimates, these two dependent measures (accuracy versus over/underestimates) can yield quite different outcomes, and signed differences cannot alone be used as an estimate of task difficulty.

Speech rate changes with chronological age and, therefore, one way to study the effects of speech rate on age estimation is to ask participants to make age estimates of voices from speakers who differ in chronological age. However, experimental research, in which the parameter of interest, in this case speech rate, is manipulated, constitutes much harder causal evidence for the effects of speech rate on age estimation. Only a few studies hitherto (Schötz, 2004; Winkler, 2007; Harnsberger et al., 2008) have studied the effect of speech rate on perceived age by actually manipulating speech rate and the study of Harnsberger et al. (2008) is most relevant as they are the only ones that study speech material longer than a few words. They reported that increased speech rate (by 20%) lowered perceived age of older speakers (74–88 years) and that decreased speech rate (by 20%) resulted in higher age estimates of middle-aged speakers although decreased speech rate did not change the perceived age of younger (21–29 years) speakers. However, Harnsberger et al. (2008) did not study the effects of increased speech rate on perception of younger speakers, nor did they study the effects of decreased speech rate on perception of older speakers. The present study will close that gap. Moreover, a change of speech rate by 20% is quite substantial and a preliminary study indicated that a manipulation of this magnitude made some voices sound “strange” according to naive listeners. No strangeness was noted when we manipulated speech rate plus minus 10% and it was therefore decided to use this smaller manipulation to see if it also had an effect on perceived age.

In sum, this study explores how subtle manipulations of the speech signal in form of a change in speech rate affect listeners’ judgment of speaker age. The effect of increased and decreased speech rate on young, middle-aged, and old voices will be analyzed. The first experiment concerns read speech while the second concerns spontaneous speech.

## Experiment 1

In Experiment 1, we investigated how a change in speech rate influenced age estimations of voices from younger, middle-aged, and older speakers. We hypothesized, extending the results from Harnsberger et al. (2008) that decreased speech rate would make all speakers sound older and increased speech rate would make all speakers sound younger, regardless of the chronological age of the speaker. Moreover, we explored whether the magnitude of this speech rate effect depends on the chronological age of the speakers.

### Method

#### Participants/Listeners

Eighty-one students (67% female) at the University of Gävle participated in the listening tests in exchange for a ticket to the movie (value of US $12). The mean age of the participants was 24 years (SD = 6.01, range 18–49 years). The studies reported in this paper were conducted in accordance with the declaration of Helsinki and the ethical guidelines given by the American Psychological Association. All participants (listeners and speakers) were adults and participated on informed consent. The listeners and the speakers signed an information agreement form. The experiment caused no harm to any part, the identity of the participants has been kept confidential, and no conflict of interest can be identified.

#### Speech Material

Voices from 36 non-smoking native speakers of Swedish were used in the study. Twelve were 20–30 years, 12 were 40–50 years, and 12 were 60–70 years. Six speakers from each age group were female and six were male. The speakers were recorded while reading a 35 word text containing written walking directions.

The recordings were made in a silent room on a computer connected to a dynamic microphone placed 15 cm from the speaker’s mouth. The recordings were edited in Audacity 1.2.6 (http://audacity.sourceforge.net). A standard feature in the program was used to compress the dynamic range of the recordings, making the loudest parts softer while keeping the volume of the soft parts the same. The threshold value was set to -12 dB and the ratio was set to 2:1. The speech samples were then normalized for intensity by setting the maximum intensity of all samples to the same value.

The manipulations of speech rate were also made in Audacity by creating two new versions of each original speech sample and decreasing the speech rate for one of them by 10% while increasing the speech rate for the other version by 10%. The pitch was kept constant for each voice across the three speech rate conditions by a standard feature in Audacity. The speech samples varied between 10 and 19 s in length after manipulation.

Average fundamental frequency for each speech sample was analyzed in Praat. As expected (e.g., Titze, 1994), men’s voices had a lower F_0_ than women’s voices as confirmed by a 2 (Gender: women, men) × 3 (Age group: young, middle aged, old) analysis of variance with F_0_ as dependent variable, *F*(1,30) = 100.16, MSE = 518.26, *p* < 0.001, $\eta_{p}^{2}$ = 0.77. However, there was no direct effect of age group or an interaction between the factors. See **Table 1** for means and variation in F_0_ over age groups and gender. Thus, F_0_ was not included as a factor in subsequent analyses.

**Table 1:** F_0_ (in Hz) of stimuli voices over age groups and gender (*M*, SD) in Experiment 1.

	Women		Men	
		
Age group	*M*	SD	*M*	SD
Young	204.84	28.64	125.73	19.06
Middle aged	202.17	27.53	127.60	15.47
Old	199.26	21.59	112.98	11.24


#### Procedure

The listening tests were conducted in a laboratory where speech samples were presented to the participants through headphones. The participants adjusted the volume to a comfortable level at the start of the experiment. They were instructed to estimate the age (in years) of each speaker they were going to hear and write their estimate in a form. Three test trials were used for familiarization with the task. A 10-s pause was set in between every speech sample. Backtracking was not allowed. In all, the experiment lasted 15–20 min.

Each participant estimated each speaker only at one speech rate. The participants were randomized into three listener groups that were balanced with regard to gender and age. Each listener group was presented to 36 speech samples (12 samples with increased speech rate, 12 with natural speech rate and 12 with decreased speech rate) in randomized order. Each set contained speech samples produced by all 36 speakers but at different speech rates. A randomized order was generated for each of the three sets of speech samples. This order was also reversed, resulting in two orders of presentation for each of the three listening groups.

#### Statistics and Design

A 3 (speaker age group: young vs. middle-aged vs. old) × 3 (speech rate: increased vs. natural vs. decreased) within-participants factorial design was used to measure differences in age estimates depending on speaker age group and speech rate. In cases of absent estimations or if listeners were acquainted with a speaker, missing values were substituted by the mean value for the particular speech sample for speaker age group, speaker gender and listener gender. This procedure was applied to 13 missing values. Two dependent measures were calculated, signed differences between age estimates and the chronological age of the target person (to investigate over- and underestimations) and the absolute/unsigned differences (to investigate accuracy) following previous studies (e.g., Vestlund et al., 2009; Voelke et al., 2012).

### Results and Discussion

**FIGURE 1:**
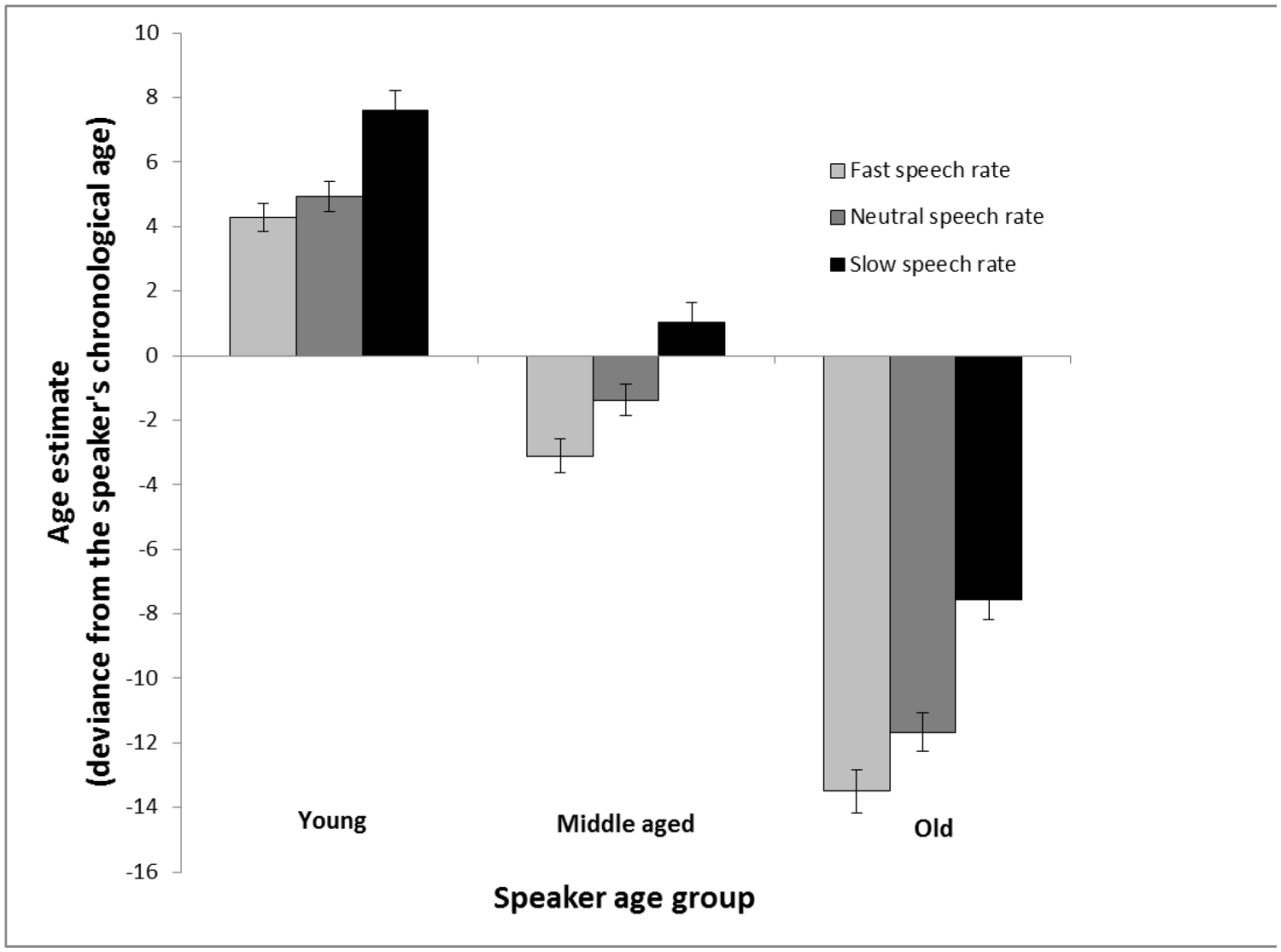
**Age estimation in Experiment 1 calculated as the average of the signed differences between the age estimations and chronological age of the speakers.** The estimates are made of voices from young, middle aged and old speakers based on recordings of read speech that are either played back at a neutral rate (same as the recording), a faster rate (10% faster), or a slower rate (10% slower). Error bars represent SEMs.

As can be seen in **Figure 1**, the age of younger speakers was overestimated (a deviation from the accurate age of the speaker above 0) and the age of older speakers was underestimated (a deviation below 0). Moreover, increased speech rate made the speaker sound younger, and decreased speech rate made the speaker sound older. This speech rate effect was most pronounced in age estimates of voices from old speakers. These conclusions were supported by a 3 (speaker age group: young vs. middle-aged vs. older) × 3 (speech rate: increased vs. natural vs. decreased) repeated measures analysis of variance. The analysis revealed a main effect of speaker age group, *F*(2,160) = 691.72, MSE = 24.26, *p* < 0.001, $\eta_{p}^{2}$ = 0.90, a main effect of speech rate, *F*(2,160) = 70.69, MSE = 17.89, *p* < 0.001, $\eta_{p}^{2}$ = 0.47, and a significant interaction between the two factors, *F*(4,320) = 2.48, MSE = 16.68, *p* = 0.044, $\eta_{p}^{2}$ = 0.03. Follow-up *t*-tests were conducted to tease apart the interaction. Fast speech rate was different from slow speech rate in age estimates of young, *t*(80) = 4.26, *p* < 0.001, middle-aged, *t*(80) = 6.83, *p* < 0.001, and old speakers, *t*(80) = 7.68, *p* < 0.001. The difference in age estimates of voices with slow and fast speech rate was larger for estimates of old speakers in comparison with estimates of young speakers, *t*(80) = 2.23, *p* = 0.029. A 2 (speaker gender) × 2 (participant gender) analysis of variance with age estimates collapsed across age groups and speech rates was computed to explore general effects of gender. It revealed that female voices are perceived as younger (*M* = -26.29, SD = 27.38) than male voices (*M* = -12.42, SD = -32.56), *F*(1,158) = 7.64, MSE = 896.08, *p* = 0.006, $\eta_{p}^{2}$ = 0.05, but yielded no effect of participant gender nor an interaction between speaker gender and participant gender.

As a control of task difficulty, the accuracy of the estimates was also analyzed. Accuracy was highest in estimations of the youngest age group (*M* = 8.10, SD = 4.29), intermediate in the middle-aged group (*M* = 9.22, SD = 3.52) and lowest in estimations of the oldest age group (*M* = 14.53, SD = 5.50). This was confirmed by a repeated measures analysis of variance with age group of target persons as independent variable (young vs. middle-aged vs. older) and accuracy as dependent variable, *F*(2,160) = 66.99, MSE = 14.23, *p* < 0.001, $\eta_{p}^{2}$ = 0.46. Estimates of young were different from middle-aged, *t*(80) = 2.07, *p* = 0.041, estimates of young were different from old, *t*(80) = 9.42, *p* < 0.001, and estimates of middle-age were different from old, *t*(80) = 9.66, *p* < 0.001.

**FIGURE 2:**
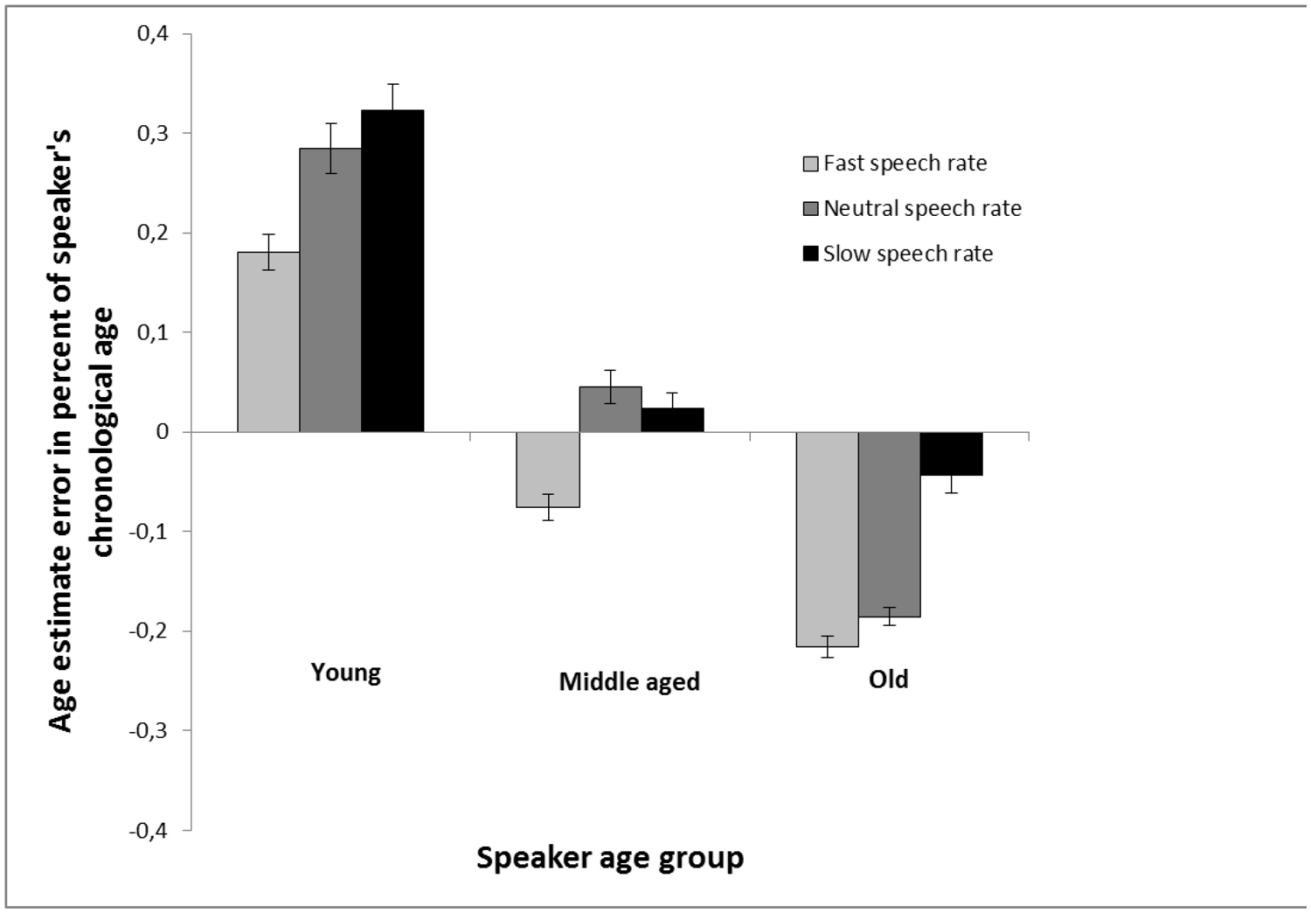
**Age estimation in Experiment 1 calculated as the percent error estimate (the average of the signed differences between the age estimations and chronological age of the speakers, divided with speakers age). The estimates are made of voices from young, middle aged, and old speakers based on recordings of read speech that are either played back at a neutral rate (same as the recording), a faster rate (10% faster) or a slower rate (10% slower). Error bars represent SEMs**.

A further control analysis was conducted in view of a “scale” problem in age estimates: For example, an estimation error of 2 years is not much (in percent) when the speaker is 65 years old, whilst an estimation error of 2 years is quite substantial when the speaker is only 4 years old. For each age estimate, respectively, the signed difference between the age estimate and speaker’s chronological age was divided with speaker’s age. Following this procedure, error estimates, expressed as percent of speaker’s chronological age, were obtained (**Figure 2**). As can be seen in **Figure 2**, which depicts percent error estimates, a speech rate effect was clearly pronounced in estimates of young speakers and old speakers, but not in middle aged speakers, and faster speech rate was overall associated with lower age estimates. A 3 (speaker age group: young vs. middle-aged vs. older) × 3 (speech rate: increased vs. natural vs. decreased) repeated measures analysis of variance with percent error estimates as dependent variable revealed a main effect of speaker age group, *F*(2,160) = 537.83, MSE = 0.02, *p* < 0.001, $\eta_{p}^{2}$ = 0.87, a main effect of speech rate, *F*(2,160) = 54.64, MSE = 0.02, *p* < 0.001, $\eta_{p}^{2}$ = 0.41, and a significant interaction between the two factors, *F*(4,320) = 8.27, MSE = 0.02, *p* < 0.001, $\eta_{p}^{2}$ = 0.09. In young speakers, faster speech rate made the speaker sound younger in comparison with neutral speech rate, *t*(80) = 3.50, *p* < 0.001, whilst the difference between slow speech rate and neutral speech rate did not reach significance, *t*(80) = 1.80, *p* = 0.075. In older speakers, there were clear cut differences between all three speech rates. Slower speech rate made them sound older in comparison with neutral speech rate, *t*(80) = 7.13, *p* < 0.001, and faster speech rate made them sound younger compared to neutral speech rate, *t*(80) = 2.80, *p* = 0.006. Taken together, the key finding from these analyses is that the speech rate effect is strongest in estimates of older speakers, but also quite strong in estimates of younger speakers, and faster speech rate makes the speaker sound younger.

The findings confirm the general assumption that speech rate is a cue to speakers’ age that listeners use as a basis for making age estimates. The effect was found for all three age groups and was not limited to middle aged and old voices as in Harnsberger et al. (2008). The interaction between speech rate and the chronological age of the speaker suggests, however, that speech rate may gain greater importance as an age cue with increased speaker age. This is shown in the analysis with regular age estimates and received some further support in the analysis of percent error estimates. The assumption that cues to speaker age are more prominent or easy to perceive in voices of younger speakers accords well with the accuracy analyses, as accuracy was higher in age estimates based on voices from younger speakers in comparison with estimates of older speakers. Thus, the listener may have to rely more on different and less informative cues when making estimates of the older and more difficult age groups.

## Experiment 2

The impact on age estimates of paralinguistic speech attributes such as speech rate is likely to depend on access to other cues such as linguistic variation, and consequently on the type of speech material to be assessed. Spontaneous speech which in contrast to read speech allows for variation in wording, should presumably yield more accurate age estimates, and age estimates of spontaneous speech should be less influenced by speech rate, compared to age estimates of read speech. Studies investigating listener’s estimation of speaker age have almost exclusively been based on speech that is produced when reading out loud (i.e., read speech) in the form of sentences, words, or vowels. From a methodological viewpoint, read speech has the advantage of control over linguistic variation and duration. Conversely, spontaneous speech should entail more variability between speech samples. However, listeners’ age estimation strategies are more likely to be based on what they have learned from their everyday interactions with others—such as the association between speech rate and the chronological age of the speaker—wherein they listen almost exclusively on spontaneous speech, not to read speech. Some evidence for this assumption has been reported in a study by Schötz (2005) who found that age estimates were more accurate when based on spontaneous speech in comparison with estimates based on read isolated words. Experiment 2 was designed to test whether speech rate is an important age cue in the context of spontaneous speech and whether it would interact with the chronological age of the speaker just as in Experiment 1. One possibility is that speech rate plays a more subordinate role as a cue to speaker age in the context of spontaneous speech, as spontaneous speech is richer in other age cues (complexity, fluency, and word selection, etc.). As in Experiment 1, accuracy served as a device to infer task difficulty.

### Method

#### Participants/Listeners

Eighty-six students (68% female) from the University of Gävle participated in the experiment in exchange for a ticket to the movie (about US $12). The mean age of the participants was 24 years (SD = 5.14, range 18–51 years).

#### Speech Material

A total of 36 original samples of spontaneous speech were used produced by the same group of speakers as in Experiment 1. The speech samples were generated by asking each speaker to provide directions on how to navigate from an origin to a destination on a map. The map represented a route taking a number of turns through an area with simple landmarks for buildings, vegetation, and water. Some speakers primarily used right–left descriptors, whereas others gave more detailed descriptions of the environment. Segments from the recordings were edited and manipulated in the same manner as in Experiment 1 using Audacity. Three versions for each speech sample were used (natural speech rate, 10% decreased speech rate and 10% increased speech rate). The duration of the speech samples before manipulation was 9–18 s.

Average fundamental frequency for each speech sample was analyzed in Praat. See **Table 2** for means and variation in F_0_ over age groups and gender. Like in Experiment 1, men’s voices had a lower F_0_ than women’s voices. This was confirmed by a 2 (Gender: women, men) × 3 (Age group: young, middle age, old) analysis of variance with F_0_ as dependent variable, *F*(1,30) = 218.02, MSE = 258.36, *p* < 0.001 $\eta_{p}^{2}$ = 0.88. There was no direct effect of age group and no interaction between gender and age group. F_0_ was therefore not analyzed further.

**Table 2:** F_0_ (in Hz) of stimuli voices over age groups and gender (*M*, SD) in Experiment 2.

	Women		Men	
		
Age group	*M*	SD	*M*	SD
Young	201.34	20.38	116.18	14.15
Middle aged	201.66	22.27	126.07	16.47
Old	193.01	8.55	116.33	9.28


#### Design and Procedure

The design and procedure was the same as in Experiment 1. The only difference was that spontaneous speech was presented instead of read speech.

### Results and Discussion

**FIGURE 3:**
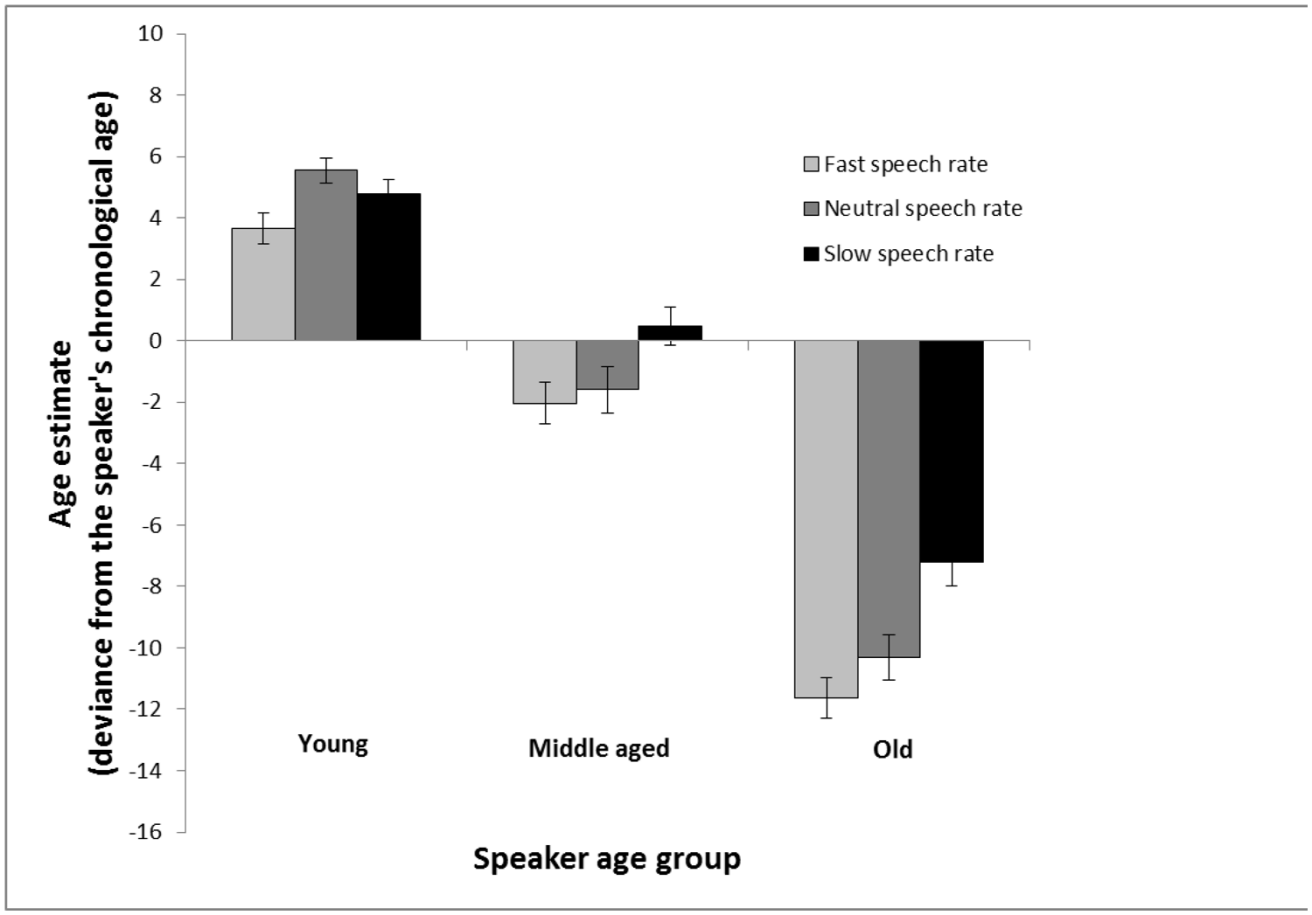
**Age estimation in Experiment 2 calculated as the average of the signed differences between the age estimations and chronological age of the speakers.** The estimates are made of voices from young, middle aged, and old speakers based on recordings of spontaneous speech that are either played back at a neutral rate (same as the recording), a faster rate (10% faster) or a slower rate (10% slower). Error bars represent SEMs.

As can be seen in **Figure 3**, the result pattern was quite similar to that found in Experiment 1. Again, the speaker sounded younger when speech rate was increased, and older when the speech rate was decreased. However, it was only in age estimates of the oldest age group that there was a clear-cut negative relationship between speech rate and age estimates. A 3 (speaker age group: young vs. middle-aged vs. old) × 3 (speech rate: increased vs. neutral vs. decreased) repeated measures analysis of variance revealed a main effect of speaker age group, *F*(2,170) = 475.64, MSE = 28.49, *p* < 0.001, $\eta_{p}^{2}$ = 0.85, a main effect of speech rate, *F*(2,170) = 22.65, MSE = 20.65, *p* < 0.001, $\eta_{p}^{2}$ = 0.21, and a significant interaction between the two factors, *F*(4,340) = 3.94, MSE = 26.53, *p* = 0.004, $\eta_{p}^{2}$ = 0.04. This interaction reveals that the effect of speech rate is linearly related to age estimates of older speakers—faster speech rate is associated with lower age estimates (i.e., faster speech rate make the speaker sound younger)—but this is not the case in estimates of young speakers—wherein highest age estimates were found for the natural speech rate. Follow-up *t*-tests showed, in estimates of young speakers, that there was no significant difference between fast and slow speech rate, *t*(85) = 1.68, *p* = 0.097, and no difference between slow and natural, *t*(85) = 1.27, *p* = 0.209, but there was a difference between fast and natural speech rate in estimates of young speakers, *t*(85) = 3.18, *p* = 0.002. However, for both middle-aged, *t*(85) = 3.31, *p* = 0.001, and older speakers, *t*(85) = 5.05, *p* < 0.001, there was a difference between fast and slow speech rate. Taken together, the speech rate effect behaves differently for the three speaker age groups. A 2 (speaker gender) × 2 (participant gender) analysis of variance with age estimates collapsed across age groups and speech rates was computed to explore general effects of gender. It revealed that men made larger underestimation errors (*M* = -29.51, SD = -31.68) compared to women (*M* = -13.18, SD = -34.05), *F*(1,168) = 9.10, MSE = 1085.49, *p* = 0.003, $\eta_{p}^{2}$ = 0.05, but yielded no effect of speaker gender nor an interaction between speaker gender and participant gender.

As in Experiment 1, the analysis of differences in accuracy between speaker age groups gave a significant main effect of speaker age, *F*(2,170) = 19.76, MSE = 20.53, *p* < 0.001, $\eta_{p}^{2}$ = 0.40, and again, accuracy was highest in estimations of the youngest age group (*M* = 6.56, SD = 3.51), lowest in estimations of the oldest age group (*M* = 11.09, SD = 5.65) and intermediate in the middle-aged group (*M* = 8.14, SD = 3.49). Estimates of young were different from middle-aged, *t*(80) = 2.07, *p* = 0.041, estimates of young were different from old, *t*(80) = 2.32, *p* = 0.023, and estimates of middle-age were different from old, *t*(80) = 4.12, *p* < 0.001.

**FIGURE 4:**
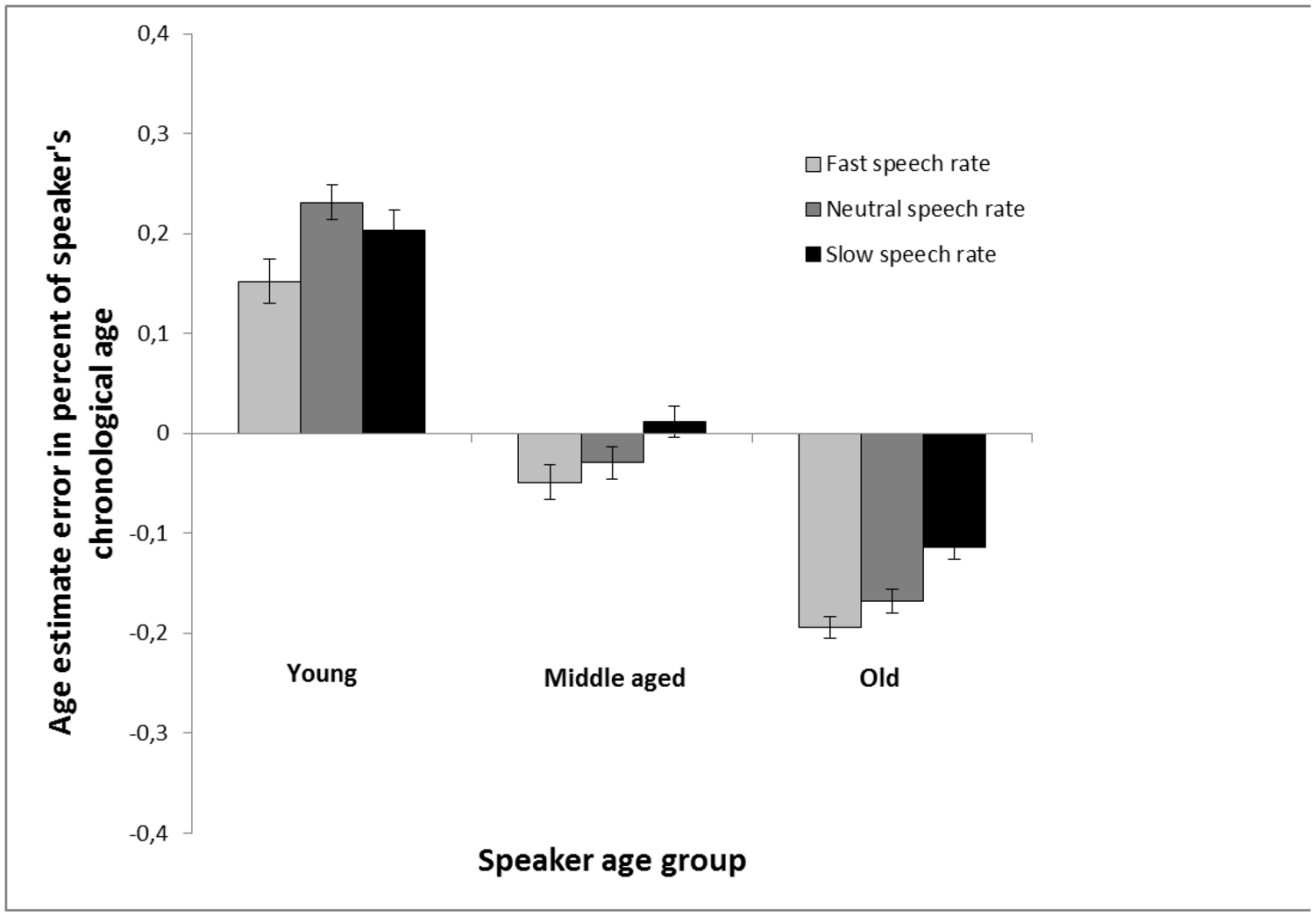
**Age estimation in Experiment 2 calculated as the percent error estimate (the average of the signed differences between the age estimations and chronological age of the speakers, divided with speakers age). The estimates are made of voices from young, middle aged, and old speakers based on recordings of read speech that are either played back at a neutral rate (same as the recording), a faster rate (10% faster) or a slower rate (10% slower). Error bars represent SEMs**.

Also, as in Experiment 1, an analysis with estimation error in percent of speaker’s chronological age was conducted. These results (**Figure 4**) were very similar to those found with regular age estimates (**Figure 3**). A 3 (speaker age group: young vs. middle-aged vs. old) × 3 (speech rate: increased vs. neutral vs. decreased) repeated measures analysis of variance revealed a main effect of speaker age group, *F*(2,170) = 464.62, MSE = 0.02, *p* < 0.001, $\eta_{p}^{2}$ = 0.85, a main effect of speech rate, *F*(2,170) = 15.56, MSE = 0.02, *p* < 0.001, $\eta_{p}^{2}$ = 0.16, and a significant interaction between the two factors, *F*(4,340) = 2.86, MSE = 0.02, *p* = 0.023, $\eta_{p}^{2}$ = 0.03. In estimates of young speakers, the difference between slow speech rate and fast speech rate did not reach significance, *t*(85) = 1.83, *p* = 0.071, and there was no difference between slow speech rate and neutral speech rate, *t*(85) = 1.12, *p* = 0.265, but fast speech rate made them sound younger in comparison with neutral speech rate, *t*(85) = 3.19, *p* = 0.002. In estimates of old speakers, faster speech rate made them sound younger in comparison with neutral speech rate, *t*(85) = 2.02, *p* = 0.046, and slower speech rate made them sound older, *t*(85) = 4.30, *p* < 0.001, and a substantial difference was found between slow and fast speech rate, *t*(85) = 5.69, *p* < 0.001.

Experiment 2 replicates the key findings from Experiment 1: listeners use speech rate as a cue to infer the age of speakers from their voices, but this cue is assigned greater weight in estimates of older speakers. When the speech is spontaneous, and hence relatively rich in age cues, the listeners seem to rely on other cues than speech rate when estimating the age of younger speakers, whilst speech rate is still an important cue in the more difficult situation of age estimates of older speakers.

### Cross-Experiment Analyses

Experiment 2 expands previous findings by showing that estimators rely less on speech rate when making age estimates of young speakers in the context of spontaneous speech compared with read speech. A cross-experiment analysis was conducted to test, within a coherent analysis, whether speech rate (slow vs. natural vs. fast) and speech material (read vs. spontaneous) interact in their effects on age estimation of younger speakers. Specifically, a visual inspection of **Figures 1** and **3** suggests that the difference between the speech rate conditions are greater for read speech than for spontaneous speech. A mixed analysis of variance with speech material as between-subject factor, speech rate as within-subject factor and over/underestimates as dependent variable was calculated to test this hypothesis. A main effect of speech rate, *F*(2,330) = 12.39, MSE = 16.64, *p* < 0.001, $\eta_{p}^{2}$ = 0.07, a main effect of speech material, *F*(1,165) = 4.17, MSE = 26.92, *p* = 0.043, $\eta_{p}^{2}$ = 0.03, and a significant interaction between the two factors, *F*(2,330) = 7.49, MSE = 16.64, *p* < 0.001, $\eta_{p}^{2}$ = 0.04, were found.

**FIGURE 5:**
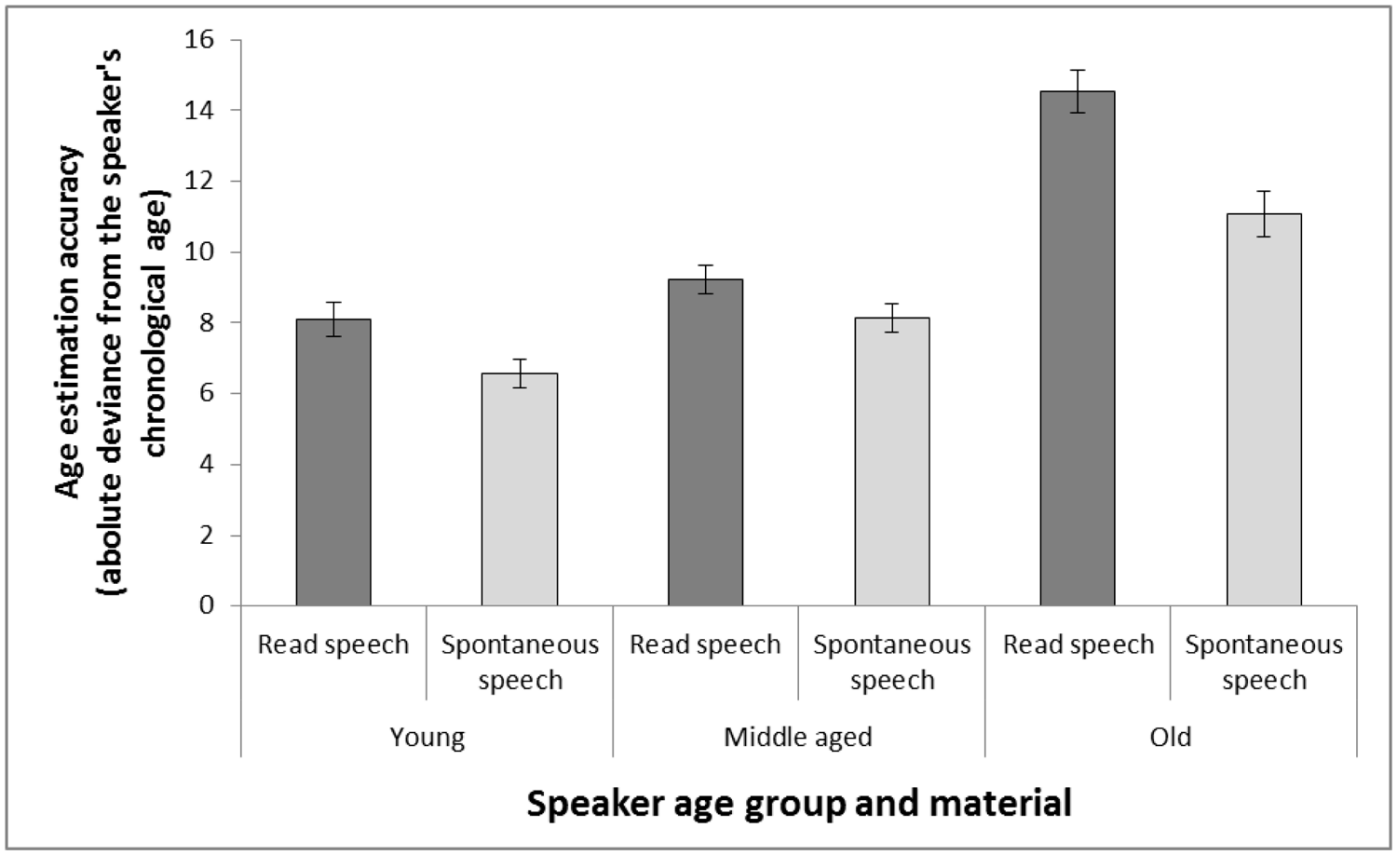
**Age estimation accuracy in Experiment 1 (read speech) and Experiment 2 (spontaneous speech). Note that lower values represent higher accuracy, as accuracy is calculated as the average of the absolute values of the difference between the age estimations and chronological age of the speakers. The estimates are made of voices from young, middle aged, and old speakers based on read speech and spontaneous speech played back at a neutral rate (same as the recording). Error bars represent SEMs**.

A cross-experiment analysis on accuracy estimates were also conducted, to test the hypothesis (of applied importance) that age estimation accuracy is higher for spontaneous speech than for read speech (**Figure 5**). A 3 (speaker age group: young vs. middle-aged vs. old) × 2 (material: read vs. spontaneous speech) repeated measures analysis of variance was performed for estimates of voices at natural speech rate from both experiments. The results supported the assumption that spontaneous speech contains more age information compared to read speech, as a main effect of speech material revealed higher accuracy in estimates based on spontaneous speech, *F*(1,165) = 19.53, MSE = 23.68, *p* < 0.001, $\eta_{p}^{2}$ = 0.11. Moreover, a significant interaction between speaker age group and material, *F*(2,340) = 4.11, MSE = 26.53, *p* = 0.004, $\eta_{p}^{2}$ = 0.04, indicated that the difference in accuracy between read and spontaneous speech was greater for the oldest age group compared to the accuracy difference due to material amongst the two younger age groups. Again, to make accurate estimates of older speakers seems to require more complex age information and may rely on different cues than what is needed to make accurate estimates of younger speakers.

## General Discussion

The experiments reported here show that speech rate is an age cue that listeners rely on when inferring the age of speakers from their voices. The current study is consistent with previous studies on speech rate (Shipp et al., 1992; Brückl and Sendlmeier, 2003; Stölten and Engstrand, 2003; Winkler, 2007; Harnsberger et al., 2008), whilst expanding those findings in several directions. Specifically, speakers are estimated as younger when they talk faster and as older when they talk slower, especially older speakers. It appears as if age estimates of younger speakers, however, are not influenced by speech rate, at least in the context of spontaneous speech wherein the speakers are free to select words as they like.

### Speech Rate as a Cue to Speaker’s Age

Harnsberger et al. (2008) found the typical speech rate effect—higher age estimates of slower speech rate and lower age estimates of faster speech rate—when speech rate was manipulated by 20%. Here, we found that a more modest speech rate manipulation of 10% produces a speech rate effect with a similar pattern. Hence, even subtle changes of speech rate can influence listeners’ perception of speaker age.

Listeners are able to distinguish between spontaneous speech and read speech (Blaauw, 1994) as they differ on several acoustic cues such as prosodic cues and spectral cues (Howell and Kadi-Hanifi, 1991; Nakamura et al., 2008). In particular, the boundaries between tone units differ between spontaneous speech and read speech (Blaauw, 1994), the position of the stresses differs and there are fewer pauses in read speech (Howell and Kadi-Hanifi, 1991) and spontaneous speech has a more constrained spectral space (Nakamura et al., 2008). Moreover, the semantic content (word choice) should be more variable between speech samples for spontaneous speech. These factors may explain why the interaction between speech rate and chronological age, in the present study, was slightly different in the context of spontaneous and read speech. Whilst the speech rate effect was quite different for spontaneous and read speech in age estimates of younger speakers, it was very similar in age estimates of older speakers. Under the assumption that acoustic factors (prosodic and spectral cues) vary in a roughly similar way between younger and older adult speakers, the reason why the speech rate effect is less pronounced in estimates of young adults is that the age of young speakers can more easily be identified from word choice. In other words, listeners may rely more on speech rate as a cue to age when making age estimates of older speakers, whereas word choice or other semantic aspects of the speech signal is used to identify the speaker as a young adult.

An additional reason for why speech rate was less influential on age estimates of young speakers is, potentially, that the listeners—who were mostly young adults—are more familiar with the way other young adults talk. This familiarity could perhaps lead to better discriminatory abilities making them able to identify a speaker as young, even when the speech signal is distorted by manipulations of speech rate. This suggestion is consistent with studies demonstrating an own-age bias in age estimates (i.e., people tend to estimate the age of others with greater accuracy when the target person is about the same age as the one making the estimate; Rhodes, 2009). Whether there is a similar own-age bias in age estimates from voices is unclear and the present study cannot provide evidence in support of this assumption, as no older listeners were included. Moreover, there was no support for an own-gender bias.

### Potential Applied Implications

Research on earwitness testimony is sparse but of applied importance as there are many situations in which voice is the most distinct and reliable cue to personal characteristics and identity, such as when the visual conditions are poor or when the face of a target is covered—conditions that are frequently found in criminal situations (Yarmey et al., 1996; Yarmey, 2001, 2004). In particular, when the crime is committed over a phone call or otherwise when a culprit’s identity can only be revealed from speech recordings, knowledge on the reliability of earwitness testimonies is quite important. One implication from the pair of experiments reported here is that speech rate should be recognized as a factor influencing the accuracy of the age estimate of the perpetrator, but only when the speaker’s age is relatively high. When the age of the speaker is relatively high, a slow speech rate would indicate that age estimates from earwitnesses are likely closer to the actual age of the culprit than when speech rate is fast. Conversely, when speech rate is fast—which arguably is the usual case in sharp earwitness situations—the age of older culprits is likely to be substantially underestimated. From an applied point of view, the higher estimation accuracy when age estimation is made on voices from spontaneous speech is also noteworthy. Estimation accuracy is underestimated when investigated in the context of read speech, a methodological aspect to consider in future studies and when drawing conclusions from extant research.

Another applied implication relates to acting. Many actors receive voice training (Werner, 1996) and may learn to use their voice to sound more male or female, for example. One implication from the present experiments is that actors may use speech rate to their advantage when attempting the sound as of a different age than they really are. A faster speech rate could make them sound younger, at least if the actor is above “young adult.”

A third potential (yet at present highly speculative) applied implication is that hearing impairments—and a corresponding hearing aid apparatus—that distort the temporal resolution of the speech signal may distort not only the reception of the speech signal and its comprehension but also other top–down cognitive speech processes such as inference of speaker age. As the effects of hearing impairments and of hearing aids co-vary with cognitive/top–down components of speech processing (Lunner et al., 2009), it is not far-fetched to assume that distortions to time resolution in speech reception can also influence a listener’s age estimation of speakers, as even slight changes in speech rate (10%) produce quite drastic changes in the listeners’ perception of the speaker’s age. A target for future research is to look into the effects of hearing aids on age estimation by voice. One possibility is that hearing aids distort F_0_ information, which could influence age estimates, just as it influences gender perception (Massida et al., 2013).

### Conclusion

Cognitive operations partake in speech processing to extract non-linguistic information from speech signals such as the age of the speaker who generates the voice. The purpose of the present paper has been to explore some of the characteristics of this rather special form of cognitive speech processing. We can conclude that speech rate is one source of information that listeners use to extract age information, especially when listening to older speakers. Speech rate is clearly not the only age cue, however, and when the speaker is relatively young and in a spontaneous speech context, the listener primarily relies on other sources of information (e.g., acoustic and linguistic).

## Conflict of Interest Statement

The authors declare that the research was conducted in the absence of any commercial or financial relationships that could be construed as a potential conflict of interest.
